# Diversification of defensins and NLRs in *Arabidopsis* species by different evolutionary mechanisms

**DOI:** 10.1186/s12862-017-1099-4

**Published:** 2017-12-15

**Authors:** Mariana Mondragón-Palomino, Remco Stam, Ajay John-Arputharaj, Thomas Dresselhaus

**Affiliations:** 1 Cell Biology and Plant Biochemistry, Biochemie-Zentrum Regensburg, University of Regensburg, Universitätstraße 31, 93053 Regensburg, Germany; 2 Chair of Phytopathology, Technical University of Munich, School of Life Sciences Weihenstephan, Emil-Ramann-Str. 2, 85354 Freising, Germany

**Keywords:** NLR, NBS-LRR, Cysteine-rich peptide, Defensin, Natural selection, Gene recombination, Molecular evolution, *Arabidopsis*, *Fusarium*, RNA-seq

## Abstract

**Background:**

Genes encoding proteins underlying host-pathogen co-evolution and which are selected for new resistance specificities frequently are under positive selection, a process that maintains diversity. Here, we tested the contribution of natural selection, recombination and transcriptional divergence to the evolutionary diversification of the plant defensins superfamily in three *Arabidopsis* species. The intracellular NOD-like receptor (NLR) family was used for comparison because positive selection has been well documented in its members. Similar to defensins, NLRs are encoded by a large and polymorphic gene family and many of their members are involved in the immune response.

**Results:**

Gene trees of *Arabidopsis* defensins (DEFLs) show a high prevalence of clades containing orthologs. This indicates that their diversity dates back to a common ancestor and species-specific duplications did not significantly contribute to gene family expansion. DEFLs are characterized by a pervasive pattern of neutral evolution with infrequent positive and negative selection as well as recombination. In comparison, most NLR alignment groups are characterized by frequent occurrence of positive selection and recombination in their leucine-rich repeat (LRR) domain as well negative selection in their nucleotide-binding (NB-ARC) domain. While major NLR subgroups are expressed in pistils and leaves both in presence or absence of pathogen infection, the members of DEFL alignment groups are predominantly transcribed in pistils. Furthermore, conserved groups of NLRs and DEFLs are differentially expressed in response to *Fusarium graminearum* regardless of whether these genes are under positive selection or not.

**Conclusions:**

The present analyses of NLRs expands previous studies in *Arabidopsis thaliana* and highlights contrasting patterns of purifying and diversifying selection affecting different gene regions. DEFL genes show a different evolutionary trend, with fewer recombination events and significantly fewer instances of natural selection. Their heterogeneous expression pattern suggests that transcriptional divergence probably made the major contribution to functional diversification. In comparison to smaller families encoding pathogenesis-related (PR) proteins under positive selection, DEFLs are involved in a wide variety of processes that altogether might pose structural and functional trade-offs to their family-wide pattern of evolution.

**Electronic supplementary material:**

The online version of this article (10.1186/s12862-017-1099-4) contains supplementary material, which is available to authorized users.

## Background

Disease resistance in plants results from antagonistic cycles of selection involving pathogen effectors and host targets. Therefore, determining how this co-evolutionary interplay may affect large gene families involved in defense responses is essential to understand their role in the evolution or abrogation of resistance. This insight might then be used to guide the development of resistant crop varieties and strategies to counteract pathogen infection.

**Fig. 1:**
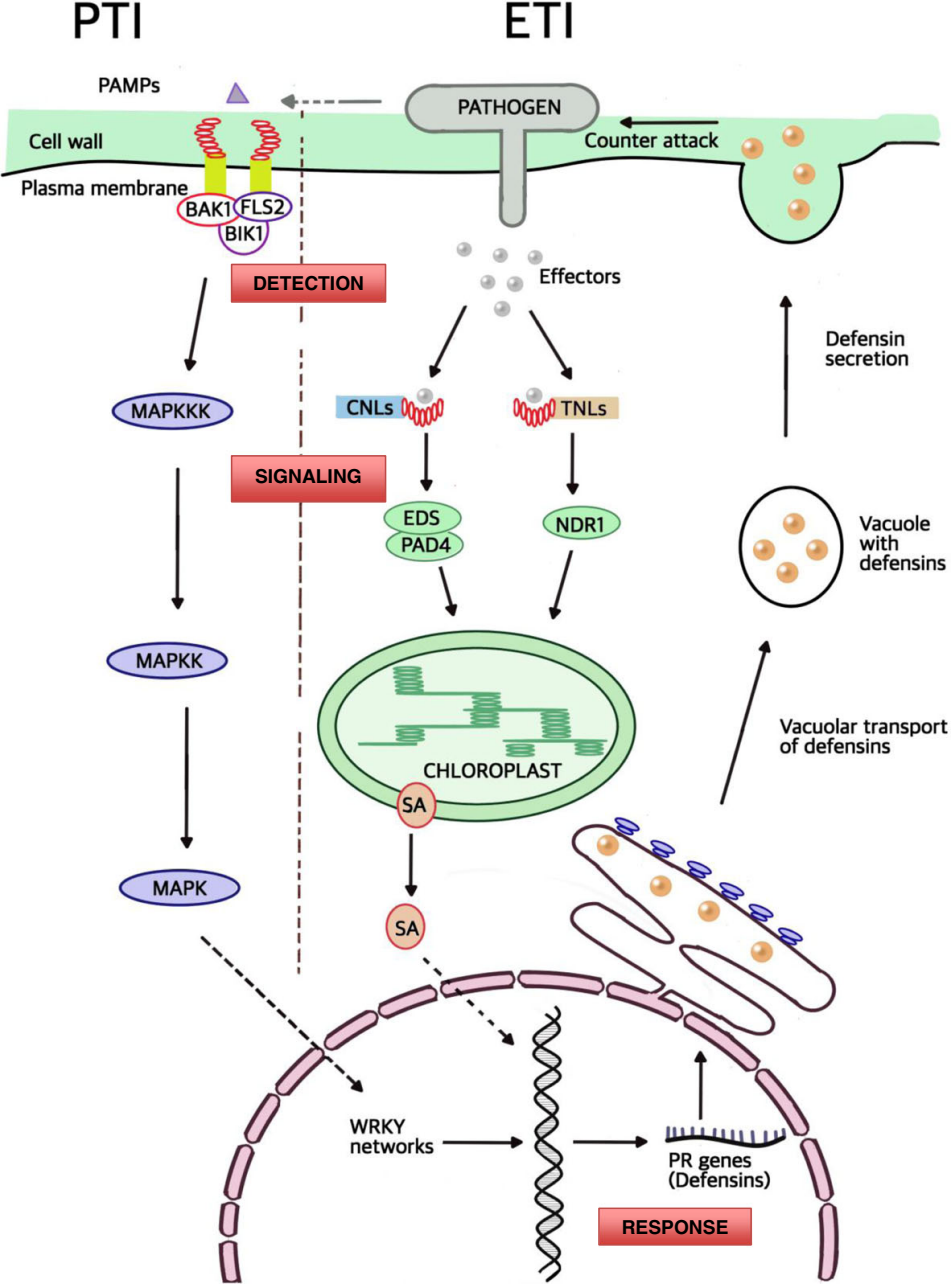
Outline of the major phases of plant immune responses. Pathogen detection can take place through direct interactions between pathogen-associated molecular patterns (PAMPs) and transmembrane receptor-like kinases RLKs or pathogen effectors and intracellular NLRs (CNLs and TNLs). After detection CNLs and TNLs trigger different signaling pathways: the pathway activated by PAMP binding involves a cascade of mitogen-activated protein kinases (MAPKs) and activation of WRKY transcription factors. A second pathway is activated by cytoplasmic proteins EDS1, PAD4, NDR1 and salicylic acid (SA). Both pattern triggered immunity (PTI) and effector triggered immunity (ETI) pathways result in a response where pathogenesis-related (PR) genes are upregulated. These include genes encoding defensins, chitinases, β-1,3-glucanases, protease inhibitors and lipid transfer proteins. As an example, transportation and secretion of defensins, which like other PR proteins have pathogen-killing activity, is indicated

Molecular evolution analyses of genes involved in pattern triggered immunity (PTI) and effector triggered immunity (ETI) have shown that those encoding proteins involved in host-pathogen interactions frequently have a higher proportion of non-synonymous to synonymous substitutions. This pattern is known as positive selection and is indicative of host-pathogen co-evolution and selection for new resistance specificities [1]. PTI is based on recognition of conserved molecular patterns like flagellin or chitin from the pathogen by receptor-like kinases (RLKs) from the host. Upon infection, pathogens suppress PTI by secreting effectors to modulate the plant defenses and establish infection. ETI is based on intracellular detection of these effector molecules by NOD-like receptors (NLRs) (Fig. 1). Signatures of positive selection identified in NLRs and RLKs, suggest that the frequent amino acid replacements favor detection of a changing variety of pathogen molecules [2, 3]. In comparison, molecules mediating the signaling pathways triggered by these receptors show little evidence of diversifying evolution, indicating that pathogen effectors generally do not target them or their evolution is functionally constrained [4], for exceptions see [5, 6]. Subsequent immune responses involves among other processes, the transcription of pathogenesis-related (PR) genes encoding a diverse group of inhibitors of pathogen growth and fitness (Fig. 1). Among them, there are several families where positive selection has been consistently detected, namely chitinases [7, 8], β-1,3-endoglucanases [9], polygalacturonase inhibitor proteins (PGIPs) [10] and thaumatin-like proteins [11]. Defensins and defensin-like genes (collectively named DEFLs) are one of the largest and most diverse PR-gene family [12]. The prevalence of positive selection in animal defensins and initial studies of some groups of plant DEFLs [13] implied a high level of polymorphism of plant DEFLs, which is also driven by co-evolution with diverse pathogen targets. However more recent studies in monocots did not find evidences of diversifying evolution [14, 15].

DEFLs are particularly intriguing because in addition to their involvement in the innate immune responses, many members of this family play key roles in reproduction, response to stress and heavy metal tolerance (reviewed in [16]). However the evolutionary mechanisms leading to this so-called functional promiscuity are not yet clear [17]. For example, DEFL SCRs are involved in self-incompatibility [18], while LUREs, another DEFL subgroup, are secreted by ovules to attract competent pollen tubes of their own species during pollination [19]. Many DEFLs of these and other subgroups are differentially expressed also in the immune responses to *Fusarium graminearum* [20]. Similarly, the *Capsicum annum* defensin *CADEF1* is expressed in response to both biotic and abiotic stresses [21] while antimicrobial PDF1s form *Arabidopsis thaliana* more closely related to DEFLs that confer heavy metal tolerance in *Arabidopsis halleri* [22].

In this study, we compare the evolutionary dynamics of DEFLs with those of NLRs with the aim of determining the relative contribution of positive natural selection, recombination and transcriptional divergence to the evolution of both families. The rationale underlying this comparison is that diversifying evolution is well documented in NLRs in *Arabidopsis* [2, 23], thus making this family an informative control and point of reference for investigating the molecular evolution of DEFLs. Both families comprise between 100 to more than 300 highly polymorphic members, many of which form clusters of tandemly duplicated genes [24, 25]. Moreover, the recently improved annotation methods for NLRs and DEFLs as well as the availability of completely sequenced genomes of several *Arabidopsis* species facilitates the comparative analyses of their molecular evolution and transcriptional divergence [12, 26, 27]. The latter process is particularly relevant to understand the diversification of both families because their members frequently transposed (moved from one position in the genome to another) frequently in the aftermath of two whole genome duplication events [28]. After transposition paralogs lost some of their *cis*-regulatory elements, which eventually might facilitate gene sub- or neofunctionalzation by transcriptional divergence.

Apart from the fact that NLRs and DEFLs are encoded by large polymorphic gene families and play key role in immune responses, they are otherwise very different. NLRs encode cytoplasmic proteins with a molecular weight over 100 kDa, they contain a nucleotide-binding domain, one or more leucine-rich repeats (LRRs) and are divided in two subclasses according to their N-terminal domain: CNLs have a coil-coil domain and TNLs possess a TIR domain. In contrast, DEFLs are small, secreted peptides with an N-terminal signal peptide and a charged or polar mature cysteine-rich domain of about 5 kDa. Three to four conserved disulfide bridges are essential to stabilize a tertiary structure comprised of an α-helix and several antiparallel β-sheets. Given the significant sequence variation of DEFLs, they have been divided in several subgroups based on their pattern of cysteines as well as other motifs [12].

Our approach to investigate the molecular evolution of DEFLs differs from previous studies in that the members of the family are further divided in subgroups of reliably aligned sequences with an identity of at least 50%. These alignment groups were tested to exclude third codon position saturation and recombination because they interfere with the detection of positive selection. We tested the contribution of transcriptional divergence to the evolution of both families by characterizing their expression in pistils and leaves infected with *Fusarium graminearum*, a heterotrophic flower-infecting fungus responsible for cereal head blight. This approach was based on the finding that DEFLs are predominantly expressed in reproductive tissues [29] as well as in response to pathogens and pollination [20].

## Results

### Annotation and phylogeny of NLRs in three *Arabidopsis* species

Among the proteins with a nucleotide-binding site (NBS), NLRs form a distinct subgroup characterized by containing a C-terminal region of leucine-rich repeats (LRRs) [30, 31]. Based on the presence of either a Toll-Interleukin-1 Receptor (TIR) homology region or a coil-coil (CC) motif in the N-terminal region, NLRs are further subdivided in two major groups known as TNLs or CNLs, respectively [32]. The presence and order of these characteristic domains guided previous annotations of NLR genes in a large number of plant species including *Arabidopsis* spp. (e.g. [25, 33, 34]). Because differences in annotation approaches hamper proper interspecific comparison of the members of a gene family, we used NLR-Parser [27] with identical settings to consistently re-annotate the set of NLRs from *Arabidopsis thaliana*, *Arabidopsis lyrata* and *Arabidopsis halleri*. NLR-Parser is based on Hidden Markov Models and has been tested for *A. thaliana* and successfully applied to other plant species [35, 36]. We repeated NLR identification in *A. thaliana* and confirmed the presence of 265 NLRs, of which 124 appear to be complete as defined by NLR-Parser, e.g. having at least a NBS, and either a CC/TIR as well as more than one LRR. It should be noted that also “partial” NLRs have been shown to be potentially functional [37] in defense signaling and therefore were included in the analysis. In *A. lyrata* we identified a total of 247 putative NLRs, which is an increase of 62 over the latest annotation [23, 38], and is closer to the number of NLRs identified in *A. thaliana*. In *A. halleri* our approach identified 208 putative NLRs. In all species, the majority of complete NLRs identified belong to the TNL class: 66% in *A. thaliana*, 63% in *A. lyrata* and 52% in *A. halleri,* respectively*.* Note that previous studies classified all genes with one or more LRR and/or one of the other domains as putative NLR and used percentage coverage as a measure of completeness, whereas NLR-Parser searches for genes with multiple domains in the right order. Genes with less than two LRR domains are annotated as partial, thus lowering the number of total genes and putative complete genes. Sequence IDs for all putative NLR in *A. lyrata, A. halleri* and *A. thaliana* are listed in Additional file 1: Data 1.

**Fig. 2:**
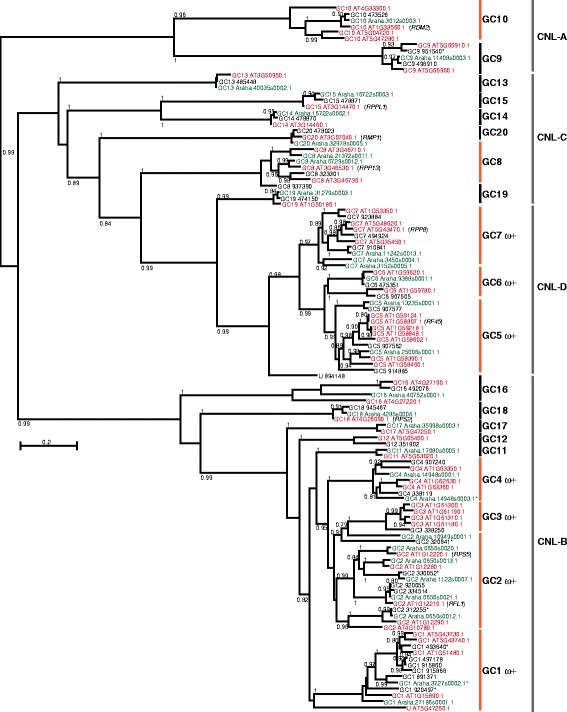
Maximum likelihood gene tree of re-annotated complete and partial CNL genes from three *Arabidopsis* species. This gene tree was inferred from the alignment of coding sequences obtained with MAFFT, where only those reliably aligned columns with a GUIDANCE2 confidence score > 0.93 were employed. The most appropriate nucleotide substitution model was selected with program SMS and the maximum likelihood phylogenies was inferred with PhyML 3.0. The numbers on every node indicate posterior probabilities >0.70 supporting the phylogenetic relationships inferred. The clades employed for defining alignment groups (GC1 to GC20) are outlined with the first column of bars on the right of the gene tree. After evaluation of similarity levels and third codon position saturation, only sequences from groups indicated with orange bars where further investigated. Among them, clades indicated with ω + reported significant evidences of positive selection. This gene tree recovered the clades CNL-A to CNL-D identified in the first published phylogeny of *A. thaliana* CNLs [25], here they are indicated with a second column of bars on the right of the gene tree. Color-coding of sequence IDs indicates *Arabidopsis halleri* in green, *Arabidopsis lyrata* in black and *Arabidopsis thaliana* in red. The names of defense genes reported in the literature are indicated in parenthesis

To assess the selective pressures on closely related NLRs, we generated so-called sequence groups using several iterative rounds of phylogenetic reconstruction as previously described [2]. In total 115 CNLs and 245 TNLs were aligned, excluding sequences that introduced gaps spanning over 25% of the length of the alignment. The resulting multiple sequence alignments were employed to infer the ML gene tree of each NLR subfamily (Fig. 2, Additional file 2: Figure S1, Additional file 3: Data 2 and Additional file 4: Data 3). As indicated in the figures, the trees inferred for both CNLs and TNLs based on alignments of members from all three species provide high statistic support to major phylogenetic clades identified by previous analyses of *A. thaliana* NLRs [25].

The overall evolutionary trend in both subfamilies consists of clades with representatives from each taxon, most of them following the phylogenetic relationships of the species: *A. lyrata* and *A. halleri* are closer to each other and *A. thaliana* is their sister group [39]. However, we identified some sequence subgroups with species-specific duplications, like GC3 and GC5 (Fig. 2) or GT5, GT6 and GT11 (Additional file 2: Figure S1).

The best-supported clades of each subfamily tree were the basis to distinguish 20 CNL and 46 TNL groups of closely related genes to investigate the molecular evolution of the family using the complete coding sequences (Table 1, Fig. 2, Additional file 2: Figure S1). In these groups, we determined that the third codon position was not saturated, the pairwise p-distance was ≤0.5 and sequences with large gaps were eliminated. The threshold value for phylogenetic distance is based on the observation that at higher values multiple substitutions often cause sequences to resemble each other by chance, thus negatively affecting the correct inference of molecular phylogenies and rates of evolution [40]. This evaluation yielded 10 CNL and 15 TNL groups with five or more sequences that were suitable for follow-up analyses (Tables 1, 2 and Additional file 5: Data 4). Of all sequence groups, 11 contain NLRs described in the literature for their role in immunity.

**Table 1:** Total number of members per gene family and species

	DEFLs	CNLs	TNLs
Members	821	115	245
*A. thaliana*	285	50	89
*A. lyrata*	315	36	100
*A. halleri*	222	29	56
Groups	156	20	46
Groups analyzed^a^	47 (47%)	9 (62%)	15 (58%)
Singletons	31	2	20

In parenthesis is the percentage of the total number of sequences comprised by these groups

^a^Only DEFL groups ≥6 sequences or CNLs and TNLs ≥5 sequences were analyzed

**Table 2:** Groups of NLRs analyzed for natural selection

Group^a^	Sequences	Length^b^	Known R-genes
GC1	9	2580	
GC2	13	2697	*RFL1, RPS5*
GC3	5	2757	
GC4	6	3213	
GC5	12	2784	*RF45, RDL5*
GC6	5	2808	
GC7	10	2871	*RPP8, RPP8L2, RPP8L3, RPP8L4*
GC8	7	2643	*RPP13, RPP13L2, RPP13L3*
GC10	5	2962	*ADR1, RDM2*
	8 (±3.12)^c^	2812.78 (±189.14)	
GT1	21	3616	*ADR2, RML1A, RML1B*
GT2	8	3171	
GT3	13	4608	
GT5	6	3957	
GT6	11	3363	
GT7	5	2754	
GT10	21	2487	*VICTR*
GT11	9	5031	*RPP4, RPP5, SNC1*
GT12	5	3168	
GT14	8	3633	*TTR1, RRS1*
GT15	7	3972	
GT19	11	3558	*RPS4*
GT21	6	3825	
GT22	6	3495	*RPP1*
GT32	5	3444	
	9.47 (± 5.27)^c^	3605.46 (±643.62)	

^a^Only groups with five or more sequences were analyzed. Group designation is arbitrary

^b^Total length of multiple sequence nucleotide alignment including positions with indels

^c^Average and sample standard deviation

### Grouping of DEFLs based on CRP classification

Cysteine-rich peptides (CRPs) are up to 170 amino acid residues in length including an N-terminal signal peptide for localization to the secretory pathway followed by a small, divergent charged or polar mature peptide domain with at an even number of conserved cysteine residues. Previously CRPs of *Arabidopsis thaliana* have been divided in groups according to sequence motif models involving distinct patterns of cysteine motifs [12]. Defensins and defensin-like peptides (DEFLs) are a class of CRPs common in all eukaryotes. Plant immature DEFL consist of about 70 amino acid residues including an N-terminal signal peptide and a charged or polar mature cysteine-rich domain. DEFLs are further characterized by four conserved disulfide bridges that stabilize a conserved tertiary structure comprised of an α-helix and several antiparallel β-sheets. Given the significant sequence variation of DEFLs, they have been divided in several CRP groups based on their pattern of cysteine residues as well as other conserved motifs [12]. In this study, we identified DEFLs of *A. lyrata* and *A. halleri* based on a previously published CRP annotation which assigned the highly diverse *A. thaliana* DEFLs to 46 CRP groups (CRP0000 to CRP1520), each of them having a specific cysteine motif [26, 29]. This approach to classify DEFLs was helpful to identify further members from *A. thaliana*, *A. lyrata* and *A. halleri* (see Methods for details). However interspecific sequence alignments comprising genes of a given CRP group includes highly diverse sequences, such that their pairwise p-distances are above the cutoff 0.5 and their third codon positions are saturated. Because these factors have negative effects on analyses of molecular evolution, we further divided the 46 CRP groups including sequences from all three *Arabidopsis* species, into smaller alignments where pairwise p-distance ≤0.5 and third codon positions are not significantly saturated (Additional file 5: Data 4). Sequence regrouping generated 46 alignments with at least six sequences each and an average length of 282 bp (Tables 1, 3 and Additional file 5: Data 4). Alignments with fewer sequences (Additional file 1: Data 1), were not considered for analyses of molecular evolution because initial runs in codeml and FUBAR showed that they rarely yielded statistically significant signals of natural selection. However, assessment of gene trees for 112 alignment groups with three or more members showed a high prevalence of clades containing sequences of the three species. Among them there were only five groups with clades comprised of species-specific duplications, most of them involving *A. lyrata* sequences. The only alignment groups with clades exclusively formed of *A. thaliana* or *A. lyrata* sequences are S1, S26 and S57 (Additional file 6: Figure S2A), which contain LUREs, well-characterized genes encoding peptides involved in species-preferential pollen tube attraction during fertilization [19]. Although some *A. halleri* sequences are part of these alignment groups, they are not part of species-specific duplications. Among other groups containing known functionally divergent genes are S119 and S120 encoding *A. thaliana* PDF1 antimicrobial defensins and heavy-metal tolerance factor *AhDEF1.3* (also known as *AhDEF1.2b*) and *AhDEF1.4* respectively. Notably, despite documented differences of gene functions, at the sequence level they are very similar and belong in clades where all three species are represented (Additional file 6: Figure S2B).

**Table 3:** Groups of DEFLs analyzed for natural selection

Group^a^	Sequences	Length^b^	CRP groups included^c^	Known DEFL-genes^d^
S117	9	267	CRP0000	*LCR76, LCR75*
S116	13	249	CRP0000	*PDF2.4, PDF2.2, PDF2.1, PDF2.3,PDF2.6*
S44	8	279	CRP0220	
S19	9	267	CRP0220	
S114	10	249	CRP0240	
S125	6	300	CRP0260	
S107	6	249	CRP0300	
S94	8	243	CRP0300	
S89	9	285	CRP0300	
S95	9	249	CRP0300	
S100	9	273	CRP0300	
S43	7	294	CRP0340	*LCR85*
S78	8	255	CRP0360	
S87	8	327	CRP0360	
S81	9	282	CRP0360	
S6	7	228	CRP0500	
S8	10	240	CRP0500	*LCR52, LCR53, LCR55, LCR56*
S132	7	240	CRP0500, CRP0560	*LCR3, LCR6, LCR20*
S130	7	315	CRP0570	*LCR11, LCR17*
S12	6	237	CRP0580	*LCR24, LCR37, LCR38*
S13	9	231	CRP0580	*LCR25, LCR26, LCR27*
S65	17	264	CRP0580	*LCR21, LCR22, LCR23, LCR35, LCR36*
S25	11	273	CRP0660	*LCR60, LCR61, LCR62, LCR63*
S17	10	396	CRP0670	*PDF3.1, PDF3.2, LCR57, LCR58*
S9	12	261	CRP0680	*LCR18, LCR39, LCR40, LCR41, LCR42*
S124	15	318	CRP0700	*ATTI1, ATTI2, ATTI3, ATTI4*
S126	6	240	CRP0770	
S42	7	261	CRP0770	
S57	7	294	CRP0810	
S1	8	357	CRP0810	
S26	9	288	CRP0810	*LURE1.2, LURE1.3, LURE1.4, LURE1.5*
S54	6	270	CRP0830	*SCRL24, SCRL25*
S63	7	315	CRP0830	*SCRL17, SCRL18, SCRL19*
S56	8	282	CRP0830	*SCRL1, SCRL2*
S62	6	291	CRP0860	*SCRL12, SCRL13*
S28	10	390	CRP0860	*SCRL4, SCRL5, SCRL6, SCRL7, SCRL8*
S122	8	270	CRP0920	
S2	6	312	CRP0940	
S70	9	276	CRP0940	
S82	6	252	CRP0960	
S73	9	264	CRP0960	
S109	6	255	CRP0980	
S71	9	309	CRP1050	
S74	6	363	CRP1100	
S68	6	294	CRP1110	
S66	7	321	CRP1120	
S119	12	258	CRP0090	*PDF1.1, PDF1.2a, PDF1.2.b, PDF1.2c, PDF1.3, AhPDF1.3*
	8.37 (±2.37)^e^	282.06 (±39.41)		

^a^Only groups with six or more sequences were analyzed. Group designation is arbitrary

^b^Total length of multiple sequence nucleotide alignment including positions with indels

^c^Groups of CRPs represented in each alignment, as defined for *A. thaliana* in [29]

^d^ *ATTI Arabidopsis thaliana* trypsin inhibitor, *CRP* Cysteine-Rich-Peptide, *LCR* Low molecular weight Cysteine-Rich Peptide, *PDF* Plant Defensin, *PR* Pathogenesis-Related

^e^Average and sample standard deviation

### Gene recombination is more frequent among NLRs compared with DEFLs

Recombination significantly contributes to the diversification of gene families. Because of their misleading effects on the detection of natural selection [41–43] we employed Geneconv and GARD (Genetic Algorithm Recombination Detection), two complementary approaches to evaluate the extent of recombination in NLR and DEFL genes [44, 45]. Geneconv identifies and scores aligned segments for which two sequences are sufficiently similar, thus indicating in the past a gene conversion or recombination event that took place in the past [44]. The highest-scoring fragments globally identified in the entire alignment are evaluated in a way similar to the BLAST method to find sequence matches in DNA or protein databases [46]. Next, these highest-scoring fragments are assigned *p*-values based on comparison with all possible fragments for the entire alignment by the method of Karlin and Altschul [47]. Subsequently, these so-called Karlin-Altschul *p*-values are Bonferroni-corrected for the number of possible sequence pair comparisons. The GARD approach is based on the premise that evolution of homologous sequences affected by recombination cannot be explained by a single phylogenetic tree, but by several - each one corresponding to every nonrecombinant fragment in the alignment [45]. GARD searches a multiple sequence alignment for segment-specific phylogenies and establishes the location of putative recombination or conversion breakpoints. These breakpoints indicate the limits of segments in an alignment that support different phylogenies. The program further assesses goodness of fit using the Akaike Information Criterion (AIC) based on a maximum likelihood model fit to [45]. Geneconv and GARD analyses provide complementary evidence on the occurrence of recombination between specific sequences and how these events globally affect the phylogenetic relationships of the sequences investigated. We collectively designate the regions identified by both methods as recombination events because we did not further evaluate whether they originated from unidirectional (gene conversion) or reciprocal recombination events.

**Fig. 3:**
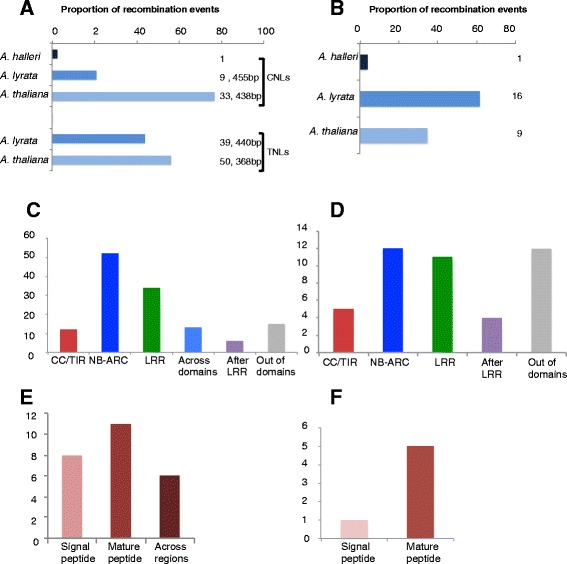
Statistically significant recombination tracks and breakpoints detected in NLRs and DEFLs. **a** Proportion of recombination tracks per species as percentage of the total number of recombination events detected with Geneconv. Numbers besides each bar indicate the actual number of recombination tracks identified in each species and their average length in each subfamily of NLRs. **b** Proportion of recombination tracks per species as percentage of the total number of recombination events detected with Geneconv in the DEFL family. Numbers besides each bar indicate the actual number of recombination tracks identified in each species in all DEFL genes analyzed. **c** Number of recombination tracks identified with Geneconv along the residues encoding three major NLR protein domains. Tracks designed as “out of domain” were identified between the boundaries of CC/TIR and NB-ARC or between NB-ARC and the LRR domain. “After LRR” indicates the amino acid residues after the last identified LRR repeat. **d** Number of breakpoints identified with GARD in the regions coding major NLR protein domains. A breakpoint indicates the beginning of a region in the multiple sequence alignment that yields a phylogeny significantly different from those based on other nucleotide positions. This phylogenetic incongruence might be due to recombination events or to significant differences in the rates of nucleotide substitution across coding sequences. **e** Number of recombination tracks identified with Geneconv along the residues encoding the signal peptide, the mature peptide or both regions encoding DEFLs. **f** Number of breakpoints identified with GARD in the regions encoding the signal or the mature peptides of DEFLs

Altogether Geneconv detected a higher number of statistically significant recombination events within NLR alignments than those detected within DEFLs (Fig. 3a and b). The largest number of events took place between CNLs and TNLs of *A. thaliana* and *A. lyrata*, frequently involving the residues encoding NB-ARC (nucleotide-binding adaptor shared by Apaf1, R genes and CED4) and LRR domains (Fig. 3c). Overall the length of these recombination tracks is not significantly different between *A. thaliana* and *A. lyrata*, the species with a higher number of events (Fig. 3a). Similarly, NLR alignments reported the largest number of statistically significant recombination breakpoints identified by GARD (Table 4, Additional file 7: Data 5), most of them also took place in the NB-ARC and LRR domains, although a larger number were also detected in-between domain regions (Fig. 3d).

**Table 4:** Statistically significant results of analysis of natural selection with codeml site models and FUBAR as well as recombination breakpoints detected with GARD for groups of NLR subfamilies CNL and TNL

Group	n^a^	LRT M1:M2	*P* ^b^	LRT M7:M8	*P* ^b^	Estimates for M8^c^	M8 BEB sites^d^	FUBAR positive^e^	FUBAR positive partitions^f^	FUBAR negative^e^	FUBAR negative partitions^f^	GARD break points^g^
CNL												
GC1	9	147.05	**<0.001**	165.85	**<0.001**	ω = 5.77; p1 = 0.05	16	26	28	72	65	2 (7)
GC2	13	22.85	**<0.001**	45.72	**<0.001**	ω = 2.00; p1 = 0.07	4	2	1	280	266	2 (7)
GC3	5	127.18	**<0.001**	129.00	**<0.001**	ω = 8.65; p1 = 0.08	36	32	30	26	22	2 (9)
GC4	6	87.78	**<0.001**	111.74	**<0.001**	ω = 4.06; p1 = 0.09	17	13	16	20	44	1 (8)
GC5	12	125.11	**<0.001**	152.85	**<0.001**	ω = 3.44; p1 = 0.08	19	22	14	145	122	5 (6)
GC6	5	50.57	**<0.001**	57.46	**<0.001**	ω = 5.17; p1 = 0.07	7	13	4	31	28	0 (7)
GC7	10	64.06	**<0.001**	108.68	**<0.001**	ω = 2.51; p1 = 0.10	19	13	13	183	170	1 (7)
TNL												
GT1	21	174.03	**<0.001**	209.33	**<0.001**	ω = 2.73; p1 = 0.07	21	3	12	316	302	1(3)
GT2	8	22.84	**<0.001**	44.70	**<0.001**	ω = 2.19; p1 = 0.1	3	11	4	137	110	1 (8)
GT3	13	218.48	**<0.001**	259.31	**<0.001**	ω = 3.10; p1 = 0.1	43	10	23	214	189	3 (5)
GT5	6	77.90	**<0.001**	90.03	**<0.001**	ω = 5.72; p1 = 0.05	17	15	13	118	83	2 (7)
GT6	11	24.74	**<0.001**	52.40	**<0.001**	ω = 1.82; p1 = 0.17	14	9	14	237	221	2 (6)
GT11	9	113.42	**<0.001**	126.25	**<0.001**	ω = 4.17; p1 = 0.07	15	5	10	79	42	1 (7)
GT14	8	34.66	**<0.001**	44.31	**<0.001**	ω = 3.09; p1 = 0.07	7	4	3	65	58	4 (7)
GT15	7	71.62	**<0.001**	82.99	**<0.001**	ω = 3.13; p1 = 0.09	21	19	4	96	63	4 (7)
GT21	6	25.63	**<0.001**	39.91	**<0.001**	ω = 2.83; p1 = 0.09	5	1	3	93	81	0 (7)
GT22	6	95.80	**<0.001**	106.74	**<0.001**	ω = 4.76; p1 = 0.07	11	32	21	71	43	5 (8)

^a^Number of sequences in the group

^b^ *P*-values are corrected for multiple comparisons with the Benjamini-Hochberg procedure employing a False Discovery Rate of 0.10. Values in bold represent significant tests in which ω is inferred to be >1.0

^c^ω is dN:dS estimated under M8; p1 is the inferred proportion of positively selected sites. Positions under positive selection are provided in Additional file 13: Data 8

^d^Number of codon position under positive selection with *P* > 0.9

^e^According to FUBAR analysis this is the number of codon positions under positive or negative selection with *P* > 0.9

^f^Results obtained with FUBAR analysis based on the partitions of all breakpoints detected with GARD regardless of their statistical significance

^g^Number of breakpoints with significant phylogenetic topological incongruence at *p* ≤ 0.1, between parentheses is the total number of breakpoints detected

N.E. means no evidence for recombination was detected. For such groups a second FUBAR analysis was not performed

Most of the few recombination events detected in DEFLs involved *A. lyrata* sequences and almost equally affected the regions encoding the N-terminal signal sequence and the mature peptide (Fig. 3e). In DEFLs, GARD detected altogether six significant recombination breakpoints (Additional file 7: Data 5), mainly in the region encoding the mature peptide (Fig. 3f).

### Differential contribution of natural selection to the diversification of NLRs and DEFLs

The programs codeml [48] and FUBAR [49] were employed to investigate the patterns of natural selection in both gene families. With codeml we compared via likelihood-ratio tests (LRTs) two pairs of so-called “site models” M1 with M2 and M7 with M8. The first model considers neutral evolution, while the second assumes a proportion of sites under positive natural selection. After FDR correction of the resulting *p*-values the evolution of most of the CNLs and TNL groups is significantly better described by both models considering codon sites under positive selection (ω > 1) (Table 4). Because the codeml approach categorizes sites into a small number of classes, which can result in misleading inference of natural selection, all datasets were also analyzed with FUBAR. This method averages over a large number of predefined site classes resulting in a practically unconstrained distribution of selection parameters and allows for the rapid identification of sites experiencing positive and negative selection. In all CNL and TNL groups where both codeml M2 and M8 were significant, FUBAR often detected a similar number of sites under positive selection (Table 4).

**Fig. 4:**
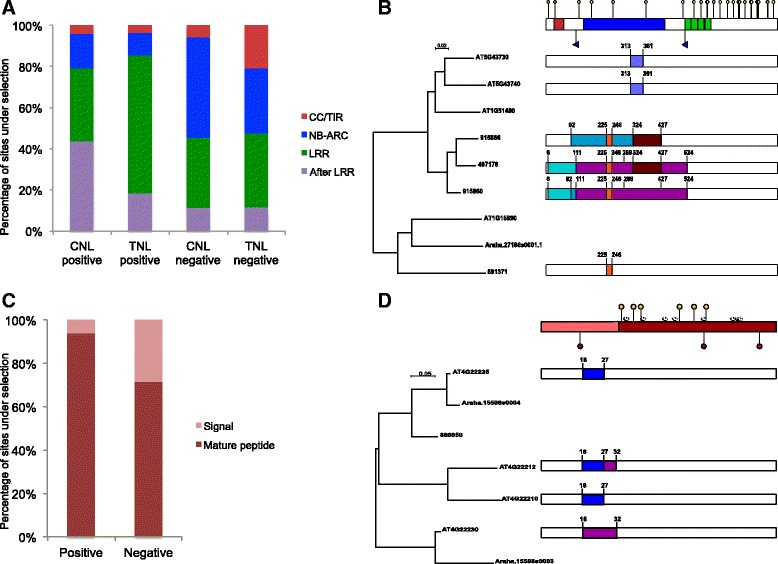
Distribution of sites under natural selection and gene conversion in domains of NLR and DEFL proteins. **a** Proportion of sites under positive or negative selection detected with FUBAR along the residues encoding the major NLR protein domains. In both CNLs and TNLs the LRR and subsequent residues are more often target of diversifying evolution, while in CNLs the NB-ARC domain reported a larger proportion of sites under negative selection. **b** Example of the distribution of statistically significant recombination tracks (Geneconv), recombination breakpoints (GARD) and sites under positive selection (FUBAR applied on partitions), in the regions encoding the coil-coil domain (red), NB-ARC (blue) and LRR domain (green) on CNL group GC1. Yellow pins indicate 28 codon positions under positive selection and purple flags identify recombination breakpoints. For the sake of clarity the 65 positions under negative selection are not indicated nor genes where conversion was not detected. Recombination tracks between two or more genes are represented with blocks of the same color. For scaling purposes recombination tracks are represented three times smaller than originally identified in nucleotide alignments. **c** Proportion of sites under positive or negative selection detected with FUBAR analyses regarding gene recombination, along the residues encoding the signal and mature peptides of DEFL genes. **d** Example of the distribution of statistically significant recombination tracks and sites under positive or negative selection on the regions encoding the signal (pink) and mature peptides (red) of DEFL group S43. Red pins indicate three codon sites under negative natural selection, yellow pins indicate six codon positions under positive selection and purple diamonds indicate positions encoding conserved cysteine residues. Recombination tracks and scaling are represented as in B

Because the detection of natural selection can be hampered by phylogenetic incongruence resulting from conversion or recombination between NLR genes, we ran FUBAR again with the corresponding phylogenies inferred for all partitions corresponding to the breakpoints detected by GARD (between parenthesis in Table 4). This approach also detected positive and negative selected codons in the same NLR groups where they were initially inferred, although the number of sites in both categories was slightly smaller (Table 4). The combined GARD-FUBAR approach showed that sites under positive natural selection are most frequently localized in the region encoding the LRR domain of TNLs and negative selected sites occur more often in the NB-ARC domain of CNLs (Fig. 4a). Because most of the groups analyzed contain known disease resistance genes (Table 2, Fig. 2 and Additional file 2: Figure S1), a reliable correlation cannot be drawn between the occurrence of positive selection and the actual involvement in the immune response of genes in those groups. Most TNL and CNL groups under positive selection belong to few, closely related and often well-supported clades (indicated in Fig. 2 and Additional file 2: Figure S1). Overall the results of gene conversion and selection analyses suggest a scenario of opposite evolutionary trends driving the evolution of NLRs: while the regions encoding the NB-ARC domain are conserved via gene conversion and/or negative selection, gene conversion and positive selection diversify the LRR domain and the region afterwards (Fig. 4a and b).

Analyses with both codeml and FUBAR show that neutral evolution prevails throughout the DEFL family. Specifically, only in eight of 48 alignments analyzed there are evidences of natural selection detected both by codeml and FUBAR (Table 5, Additional file 7: Data 5). Alignments with evidences of positive selection belong to diverse CRP groups, including three with known LCRs (Low-molecular weight, Cysteine-Rich genes) and SCRLs (*S* locus cysteine-rich-like genes), however they do not consistently contain experimentally characterized genes. In these groups the number of codon sites under positive selection detected by both codeml and FUBAR is relatively small and more frequent in the region encoding the mature peptide (Fig. 4c and d). Because of the lower occurrence of gene conversion and recombination in this family, there were few groups with GARD recombination breakpoints reanalyzed for positive and negative selection with FUBAR (Additional file 7: Data 5). In this case, the number of sites detected did not change significantly from those initially reported (Table 5).

**Table 5:** Significant results of analysis of natural selection with codeml site models and FUBAR as well as recombination breakpoints detected with GARD for groups of DEFL genes

CRP groups^a^	Group	LRT M1:M2	*P* ^b^	LRT M7:M8	*P* ^b^	Estimates for M8^c^		M8 BEB sites^d^	FUBAR positive^e^	FUBAR positive partitions^f^	FUBAR negative^e^	FUBAR negative partitions^f^	GARD break points^g^
CRP0260	S125	12.03	**0.03**	12.08	**0.04**	ω = 4.92	p1 = 0.2	5	4	3	2	2	0 (3)
CRP0340	S43	44.01	**0.01**	24.85	**0.01**	ω = 5.42	p1 = 0.22	8	6	6	3	3	0 (1)
CRP0360	S81	16.99	**0.01**	19.27	**0.01**	ω = 5.66	p1 = 0.16	3	2	3	3	2	0 (2)
CRP0360	S87	38.60	**0.01**	44.39	**0.01**	ω = 5.25	p1 = 0.28	14	4	6	2	2	0 (2)
CRP0560	S14	22.32	**0.01**	10.52	**0.05**	ω = 3.25	p1 = 0.53	7	3	3	2	2	0 (4)
CRP0580	S65	7.64	**0.03**	9.57	**0.06**	ω = 3.66	p1 = 0.08	2	2	N.A.	3	N.A.	N.E.
CRP0830	S63	9.30	**0.10**	9.52	**0.06**	ω = 9.65	p1 = 0.04	3	7	8	3	4	0 (2)
CRP1110	S68	16.57	**0.01**	16.68	**0.01**	ω = 5.45	p1 = 0.16	7	5	5	2	4	0 (1)

^a^Cysteine Rich Peptide groups (CRPs) of defensin and defensin-like genes (DEFLs) as defined for *A. thaliana* in [29]

^b^ *P*-values are corrected for multiple comparisons with the Benjamini-Hochberg procedure employing a False Discovery Rate of 0.10. Values in bold represent significant tests in which ω is inferred to be >1.0

^c^ω is dN:dS estimated under M8; p1 is the inferred proportion of positively selected sites. Positions under positive selection are provided in Additional file 13: Data 8

^d^Number of codon position under positive selection with M8 inferred with Bayes Empirical Bayes analysis with *P* > 0.9

^e^According to the FUBAR procedure this is the number of codon positions under positive or negative selection with *P* > 0.9

^f^Results obtained with FUBAR analysis based on the partitions of all breakpoints detected with GARD regardless of their statistical significance

^g^Number of breakpoints with significant phylogenetic topological incongruence at *p* ≤ 0.1, between parentheses is the total number of breakpoints detected. N.E. means no evidence for recombination was detected. For such groups a second FUBAR analysis was not performed (N.A.)

### Generation and quality of RNA-seq data from pistils and leaves of *Arabidopsis* species infected with *Fusarium graminearum*

All experimental and control treatments of pistil samples were performed 24 h after flower emasculation (Additional file 8: Figure S3). Specifically, pistils and cauline leaves of *A. halleri*, *A. thaliana* and *A. lyrata* were inoculated by dipping and spraying with *F. graminearum* conidia solution and incubated for 3 days in a moist chamber under long day conditions. This inoculation approach ensured effective *Fusarium* infection and avoided artifactual responses (e.g. through incubation in the dark or leaf infiltration with a syringe). As a control, pistils collected 24 h after emasculation, but otherwise untreated, were used for comparison with infected pistils. The surface of infected pistils and leaves collected 3 days after infection (3 DAI) showed profuse hyphal growth in *A. lyrata* (Additional file 8: Figure S3) as well as in *A. thaliana* and *A. halleri* [20]. For both control and treatments, pistils or leaves from three biological replicates were collected, each replicate containing equivalent amounts of material from 4 individual plants.

Total RNA from pistils infected with *Fusarium* was employed to compare the dynamics of gene expression patterns with that of untreated pistils. RNA sequencing yielded reads with a mean Q quality score ≥ 36 for over 94% of the reads in all biological replicates (Table 6), indicating that the base call accuracy of sequencing was well above 99.9% [50]. After quality control and trimming, reads were mapped to the re-annotated version of the *A. lyrata* genome. Table 6 summarizes the most important aspects of RNA sequencing and mapping for *A. lyrata*. The results corresponding to *A. thaliana* and *A. halleri* have been published recently [20] and are presented in Additional file 9: Data 6. Reproducibility of RNA-seq results was confirmed with qPCR assays on a set of fourteen candidate DEFL genes (Figure S2 and Figure S3 in [20]). Correlation analysis of this data showed a R^2^ = 0.83 between the log_2_ fold changes detected by RNA-seq and qPCR [20].

**Table 6:** Characteristics of the *A. lyrata* transcriptomes sequenced

Conditions compared	Total reads	Percent of ≥ Q30 Bases	Mean Quality Score	Mapped reads	Genes expressed^a^
*A. lyrata* pistils untreated	96,737,148	94.88	36.69	82,058,723	67.49
*A. lyrata* pistils infected	117,134,804	94.09	36.40	89,328,582	62.79
*A. lyrata* leaf untreated	123,196,130	95.34	36.81	104,112,543	58.43
*A. lyrata* leaf infected	92,065,932	94.89	36.65	77,815,224	58.74

Genes expressed are those with a RPKM ≥ 1

^a^ As percentage of the 29,675 genes included in the reannotated genomes of *A. lyrata* V 2.1, [77]

To maintain similar levels of variation between the biological replicates investigated, principal component analysis (PCA) and box plots were employed to select the two most similar of three biological replicates initially obtained for each experimental and control condition. Differential gene expression analysis was based on read counts from infected pistils and leaves compared to those obtained from untreated tissues, respectively. Differentially expressed genes (DEGs) were those with a false discovery rate-corrected *p*-value below 0.0005 and an expression fold change ≥2 (upregulation) or ≤ − 2 (downregulation), and which are expressed with at least one read per kilobase of transcript per million mapped reads (RPKM). The lists of DEGs in each comparison as well as their corresponding fold change and RPKM values are provided in Additional file 10: Data 7. Pistil and leaf infection resulted in a similar percentage of expressed genes (Table 6). The proportion of DEGs was in the same ranges as previously obtained for *A. halleri* and *A. thaliana* (Additional file 9: Data 6). In this study, we compare the patterns of expression and differential gene expression of NLRs and DEFLs in the context of their phylogenies and molecular evolution.

### Contrasting expression patterns of NLRs and DEFLs in response to *Fusarium graminearum* pistil and leaf infection

The pattern of RNA-seq expression of NLRs and DEFLs in control and *Fusarium graminearum* infected pistils and leaves contrasts with the above described patterns of recombination and molecular evolution. About 62% of CNLs and 44% of all TNLs from the three species are expressed (with at least one read per kilobase of transcript per million mapped reads - RPKM) at all four conditions studied.

**Fig. 5:**
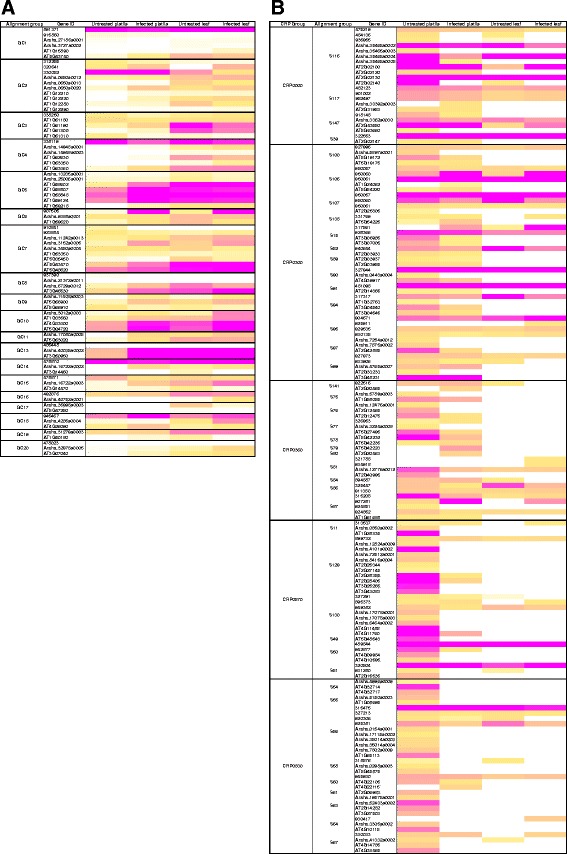
Heat map of CNLs and DEFL gene expression in untreated and *Fusarium* infected pistils and leaves in *Arabidopsis* species. **a** CNLs. **b** DEFLs. All genes with average expression signals >1 RPKM in at least one condition are shown. Gene expression was scaled based on the distribution of all expression values for each gene family. White color denotes no expression, yellow means expression values in the 50th percentile (intermediate) and magenta denotes expression values in the 90th percentile (high). Due to space limitation, we present here only the largest five CRP groups of DEFL genes. In Additional file 12: Figure S5 all expressed members of this family are shown

Visualization of the patterns of expression shows that although closely related NLR sequences from the same group might be expressed at all conditions, the actual number of reads can vary widely within and between species (Fig. 5a and Additional file 11: Figure S4, Additional file 10: Data 7). However, in both TNL and CNL classes several of the groups with highest average expression in infected pistils are also among those most highly expressed in infected leaves. Although several of the highest expressed TNL and CNL groups also display significant positive selection and might include known disease resistance genes, this trend was also detected in groups with intermediate and lower expression levels suggesting that there is no correlation between positive selection and higher levels of expression during *Fusarium* infection (Additional file 10: Data 7).

**Fig. 6:**
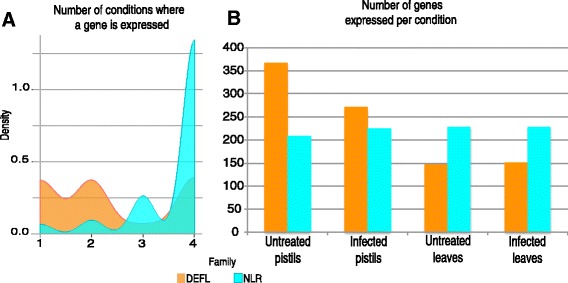
Distribution and frequency of DEFL and NLR gene expression in the conditions tested. **a** The density plot illustrates the distribution of genes expressed on different numbers of conditions here investigated. The peaks indicate that most genes of the DEFL family are expressed in one or two conditions, while NLR genes are predominantly expressed in all four conditions. These trends suggest that in the course of evolution the members of the DEFL family significantly diverged at the regulatory level in comparison to NLRs. **b** The bar chart represents the number of members from the DEFL or NLR family that are expressed in pistils or leaves under different conditions. While DEFLs are predominantly expressed in pistils, a similar number of NLRs are consistently expressed in all four conditions. These different patterns highlight the regulatory divergence among the members of the DEFL family

In contrast, fewer DEFLs are expressed at examined conditions and generally display lower transcript levels compared with NLRs (Fig. 5b, Additional file 12: Figure S5, Additional file 10: Data 7). Only 15% of DEFLs from all three species are expressed at all conditions studied, while 43% are not expressed above the 1 RPKM threshold. In this context, most DEFL groups are characterized by genes with widely divergent patterns and levels of gene expression, regardless of their pattern of molecular evolution or recombination (Fig. 5b, Additional file 12: Figure S5). The transcriptional divergence between closely related DEFLs is particularly clear when considering the distribution and frequency of expression in the conditions tested (Fig. 6). DEFL gene expression was detected predominantly in one or two conditions (Fig. 6a), most frequently in pistils untreated or infected (Fig. 6b). In contrast, the consistent expression of NLRs in all four conditions tested suggests their family-wide patterns of regulation did not strongly diverge in the course of *Arabidopsis* evolution.

#### Differential gene expression

**Fig. 7:**
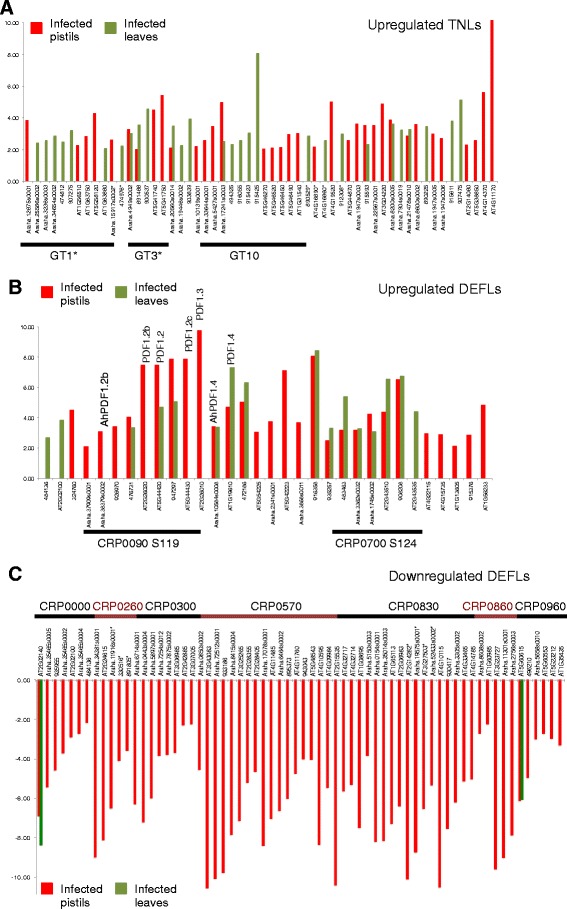
Divergence and conservation of differential gene expression of TNLs and DEFLs in *Arabidopsis* pistils and leaves infected by *Fusarium graminearum*. **a** Most differentially expressed TNLs in the species analyzed are significantly upregulated in infected leaves or pistils of the *Arabidopsis* species investigated. A large proportion of the genes upregulated belong to three top largest subgroups of genes in this family: GT1, GT3 and GT10. Asterisks indicate groups with significant positive selection. Additional file 10: Data 7 provides further details of other genes under selection and upregulated during *Fusarium* infection. **b** DEFLs differentially expressed in response to *Fusarium graminearum* are often upregulated in both pistils and leaves. A large proportion belongs to S119 and S124, two relatively large groups with representatives from all species investigated. On top of the corresponding columns the names of DEFL genes from *A. thaliana* and *A. halleri* are indicated, which are discussed in the text due to their involvement in pathogen killing and heavy metal resistance. **c** The largest group of differentially expressed DEFLs in Arabidopsis involves those downregulated during pistil infection. Although these genes belong to a variety of different groups, a large proportion of them are members of CRP0570 and CRP0830

Analysis of differential gene expression between *Fusarium* infected tissues and untreated samples showed that 19% and 22% of CNLs and TNLs from all three species were significantly upregulated both in pistils and leaves, respectively (Fig. 7a and Additional file 10: Data 7), while almost none of them were downregulated. Over half of the upregulated TNLs belong to three closely related gene groups GT1, GT3 and GT10, the first two reported significant positive selection. These results illustrate well the high degree of transcriptional divergence even between closely related members from each family (Fig. 7a).

Similar to NLRs, about 16% of all DEFLs are differentially expressed in response to *Fusarium* infection. Upregulation occurs in pistils and leaves, predominantly involving genes from groups S119 and S124 (CRP00090 and CRP0700), some with increased expression levels in both tissues tested (Fig. 7b). In contrast, significant DEFL downregulation of many DEFL groups takes places in *Fusarium* infected pistils and involves the largest proportion of differentially expressed DEFLs (Fig. 7c). Although members of diverse CRP groups from all three species are involved, the largest number of genes belongs to CRP0570 and CRP0830 (Fig. 7c). This result corroborates and broadens previous results on *F. graminearum* infected pistils of *A. thaliana* and *A. halleri* [20].

## Discussion

### Re-evaluating positive and negative selection in NLRs

We re-annotated NLR genes in three *Arabidopsis* spp. and detected significantly more NLR candidate genes compared to a previous study (265 and 247 against 159 and 185 in *A thaliana* and *A. lyrata*, respectively) [38]. This study applied the same annotation method on all three species and thus allowed better comparisons. We found slightly lower numbers of NLR in *A. halleri* compared to the other two species. However, this could be the outcome of the more recent annotation of this genome. In addition, the repetitiveness of regions encoding LRRs are more difficult to assemble than other, more regular genetic loci. Similarly as [38] observed in their *A. thaliana / A. lyrata* phylogenies, our sequence groups contain in most cases, members from all three species. This allowed us to reexamine codon-based selective pressure as well as recombination events and thus expanding on previously performed evolutionary analyses [2, 38]. The gene trees obtained with the re-annotated sequences do not significantly differ from the first studies of *A. thaliana,* suggesting that despite major differences in genome size, habitat and life cycle of these three species, major taxon-specific clades have not evolved.

When comparing all members of the NLR family within *A. thaliana*, positive selection has been shown to be a major force shaping their diversity. About 50% of the analyzed NLRs, divided over 10 sequence groups, showed sites under positive selection. 70% of these sites occur in the LRR region of the genes [2]. Expansion of these analyses by including *A. lyrata* showed that still about 50% of R genes showed positive selection. In addition, presence/absence polymorphisms of a large number of gene orthologs are evident between both species [23]. Such findings backed up the consensus that NLR genes are likely to evolve rapidly and show a clear “birth and death” pattern where alleles rapidly appear and disappear [51].

We extended these analyses to other well-known members of the genus and included additional quality controls of the alignments employed. Highly divergent gene families are prone to codon saturation, which can mislead the detection of positive selection. Specifically, we checked for third codon position saturation, levels of sequence divergence as well as more detailed analysis of possible recombination events. For this purpose, we employed the phylogenies for all partitions detected by GARD to investigate the occurrence of selection with FUBAR. Interestingly, comparing the results of FUBAR with those yielded by the combined GARD-FUBAR approach, shows that considering possible recombination events did not greatly change the outcome of the selection analyses.

Overall the analysis of NLRs showed that recombination and negative selection preserve the sequence and integrity of the NB-ARC domain, while recombination in the presence of prevailing positive selection re-assorts variation in the LRR domain and post LRR region. These distinct patterns of evolution reflect recent findings on the structural basis of NLR function. A model based on the properties of the different domains proposes that in absence of pathogen effectors the interaction of the NB-ARC domain with the N-terminal part of the LRR maintains the closed conformation of the protein [52]. Thus the prevalence of purifying selection and recombination or gene conversion in the NB-ARC domain, but also in part of the LRR domain reflect the role of these regions in preserving the stability of NLR protein folding and autoinhibition. In contrast, the C-terminal end of the LRR domain, encoded by the region were positive selection prevails, is exposed, senses charge changes in its environment and releases autoinhibition upon pathogen perception. The conformational changes triggered by the LRR allow the NB-ARC domain to exchange ADP for ATP necessary for downstream signaling [52].

### Evolution of DEFLs

Early studies on mammalian α-defensin evolution showed evidences of positive selection in the sequence regions encoding mature peptides [53]. These observations suggested that gene duplications in the defensin family were often followed by a rapid burst of positively selected amino acid changes leading to functional differentiation of paralogs [53]. This scenario received further support from comprehensive studies based on ML models that demonstrated the occurrence of positive selection in specific residues of the mature peptide of α-defensin [54–56]. Although many plant DEFLs are also involved in pathogen inhibition and killing they are not evolutionarily related to animal α-defensins [57]. Plant DEFL families have more members compared with vertebrates and these are on average longer and more diverse. Although both groups have similar amino acid biases they generate different tertiary structures and pattern of conserved disulfide bridges [57].

Although ratios of the counts of non-synonymous to synonymous substitutions in *Arabidopsis* DEFLs suggested that positive selection diversifies specific regions encoding the mature peptide [13], a more recent analysis of grass DEFLs suggests otherwise. Specifically, analysis of the ratio of synonymous to nonsynonymous substitution *Ka*/*Ks* between genes residing in duplicated grass genomic regions showed that the family is subject to purifying selection, while a sliding window analyses detected some regions evolve under positive selection [15]. However, the latter approach has been shown to produce artifactual trends of synonymous and nonsynonymous rate variation and is invalid because it does not correct for multiple testing [58]. More recently, a study on the evolution of CRPs in six closely related *Oryza* genomes reported a *dN*/*dS* ratio < 1 for all pairwise combinations of concatenated defensin coding sequences concluding that positive selection did not occur within CRPs [14]. In comparison, the approach we employed was based on reliable alignments of moderately divergent coding sequences, thus avoiding third codon position saturation, one of the major caveats preventing reliable evolutionary analysis of this family. Moreover, in contrast to previous work, both codeml and FUBAR yield detailed information regarding the occurrence of positive and negative selection as well as the codon sites affected by such trends. The fact that our results were corrected for multiple comparisons and consistent between methods adds further support to our analysis strategy. In the present analysis, a series of codon-based ML tests showed that although eight DEFL groups reported statistically significant evidences of positive and negative selection, a substantial proportion of the family in the genus *Arabidopsis* is subject to neutral evolution. While these results do not exclude that diversifying evolution takes place in some gene lineages for a short period of time, it is obvious that in terms of their molecular evolution, plant defensins are not under a prevalent regime of diversifying selection like animal α-defensins. This observation further supports the notion that they are not evolutionarily related and their roles in the immune response are different although their overall structures converged during evolution [57].

In plants and animals high immune gene diversity can confer a selective advantage to hosts facing rapidly evolving and diverse pathogens. In the case of plant defensins gene and genome duplication generated a large and polymorphic reservoir of antimicrobial factors that undergo rapid turnover in number and structure [12, 14, 24]. *Arabidopsis* DEFLs are characterized by a high proportion of gene family variation in length and pattern of conserved cysteines [12]. This information in conjunction with our results suggests that ancient events of gene duplication in their common ancestor as well as insertions and deletions played a more important role in their diversification as a family, compared to diversifying selection.

A major aspect playing a role in the evolution of DEFLs is their diversification of expression pattern. RNA-seq analyses revealed that even closely related genes show different levels and patterns of expression during fungal infection. The collective downregulation of DEFL genes observed in pistils might be a conserved mechanism employed by *Fusarium* to inhibit the immune responses.

### Species-specific divergence of DEFLs and emergence of novel functions

Our analyses further showed that *Arabidopsis* DEFLs form a very large and divergent gene family formed by small groups of similar sequences detected in all three *Arabidopsis* species and thus possibly existed already in their common ancestor. This large number of very different ortholog groups has been retained at least since the divergence of the *Arabidopsis* species occurred some 7–10 MYA [28] and contains few species-specific duplications and singletons. Assuming pathogen growth inhibition and killing is the ancestral role of DEFLs, we hypothesize that this large number of diverse DEFL genes was initially preserved because they provided a selective advantage to counteract pathogens. Subsequently, the functional divergence of this family has been facilitated by ancient and recent transposition events following WGD events that possibly diversified the regulatory properties of DEFLs and led to their preservation [28].

Two examples that illustrate the widely different ways in which DEFLs diverge functionally are LUREs and PDFs. LUREs are rather exceptional DEFLs, because they consistently formed groups of highly similar, species-specific duplicates (Additional file 6: Figure S2A). LURE genes encode peptides that are essential in the species-preferential attraction of pollen tubes towards ovules during double fertilization [19]. While their interspecific divergence might have involved episodic diversifying evolution, their current role as messengers between gametophytes acting at multiple redundant RLKs [59, 60] probably constrains their inter-specific divergence as suggested by their short branch lengths (Additional file 6: Figure S2A). However, functional divergence in DEFLs might evolve without the occurrence of species-specific duplications and/or positive selection. Specifically, while closely related PDF1s of *A. thaliana* are important for pathogen killing, their *A. halleri* orthologs AhDEF1.3 (S119), AhPDF1.4 (S120) and AhPDF1.5 are involved in heavy metal tolerance and have antifungal in vitro activity against *Fusarium oxysporum* [22]. Their high level of similarity and the fact that both *A. thaliana* and *A. halleri PDF1* genes are also differentially expressed during *Fusarium* pistil infection (Fig. 7b) supports the notion that these peptides adopted different and sometimes even simultaneous multiple roles during evolution [22]. The hypothesis that during evolution transcriptional divergence in location and levels of expression has been more important in the functional diversification of DEFLs compared to natural selection is well exemplified by *A. thaliana* and *A. halleri* PDFs. The fundamental difference between these groups of DEFLs is that there is a higher constitutive accumulation of *PDF1s* in *A. halleri* in both shoots and roots in comparison with *A. thaliana*, where these genes are not expressed in roots [22, 61]. The high degree of conservation and the significant occurrence of purifying selection in group S119 suggest the evolution of *PDF1*s and *AhDEF1*s might be constrained by their multiple roles. Further functional characterization of the DEFL family will elucidate the extent of functional promiscuity.

## Conclusions

The analysis of NLRs and DEFLs allowed comparing the selective pressure of genes with different functional properties in detection and response to ETI and PTI (Fig. 1). The present analyses of NLRs confirms previous studies in *Arabidopsis thaliana* and highlights contrasting patterns of purifying and diversifying selection affecting the NB-ARC and LRR/post-LRR regions, which might be explained by the different structural properties of these domains. Although positive selection has been detected in gene families encoding inhibitors of pathogen growth and fitness, like chitinases [7, 8], β-1,3-endoglucanases [9], polygalacturonase inhibitor proteins (PGIPs) [10] and thaumatin-like proteins [11], we detected significant evidences of positive selection only in a relatively small fraction of *Arabidopsis* DEFLs. Their expression pattern, however, suggests that, compared with positive selection, transcriptional divergence probably made a more important contribution to DEFL diversification. This regulatory divergence was possibly the outcome of frequent genomic transposition [28]. In comparison with smaller families of pathogenesis-related genes under positive selection, DEFLs and other CRPs appear to be involved in diverse biological processes besides defense and act, for example, as signaling ligands during fertilization processes, development of reproductive structures, heavy metal resistance. These heterogeneous functions and interactions with cell surface receptors and channels might altogether pose different structural and functional constraints to frequent substitutions, thus resulting in a family-wide pattern of neutral evolution.

## Methods

### NLR re-annotation

To assure consistent comparison we re-annotated all NLR genes in *A. thaliana,* and additionally in *A. lyrata* and *A. halleri* using NLR-Parser [27]. We obtained genome sequences for *Arabidopsis thaliana* (TAIR10) from arabidopsis.org, used *Arabidopsis lyrata* v.1.07 and *A. halleri* v.1.1 from Phytozome (https://phytozome.jgi.doe.gov/). MEME-suite [62] and NLR-Parser [27] were employed to re-annotate NLR genes in *Arabidopsis* species by identifying presumed NB-ARC domains of each protein. MEME-suite [62] was run with high *p*-value cut off of 10 using training sets on the predicted proteins to identify all NLR motifs in the three genomes. All motif-containing sequences and used NLR-Parser (default settings, e-value cut off 1 × 10^−6^) were extracted to annotate putative NLRs and their subdomains. The longest ORF was selected when multiple splice variants were identified; if they had equal length, the first variant was taken.

### NLR phylogeny reconstruction and grouping

To reconstruct a reliable alignment for inferring the NLR phylogeny, we employed only sequences that contained a N-terminal domain (coiled-coil or TIR) and at least one NB-ARC and LRR domains. Coding sequences of all CNLs and TNLs were identified and separately aligned as codon sequences with MAFFT [63] implemented in the GUIDANCE2 server [64] with a maximum of 50 iterations and 100 alternative guide trees and the 6mer pairwise alignment approach (Additional file 3: Data 2a and Additional file 4: Data 3a). The reliability of the resulting alignments was subsequently evaluated with the GUIDANCE2 algorithm [65]. In both alignments of CNLs and TNLs, all columns with a GUIDANCE2 confidence score > 0.93 were employed as they are considered reliably aligned (Additional file 3: Data 2b and Additional file 4: Data 3b). The most appropriate nucleotide substitution model for each multiple sequence alignment was selected with program SMS implemented in PhyML 3.0 [66]. Models were selected based on the Akaike Information Criterion (AIC). Subsequently maximum likelihood phylogenies were inferred with PhyML 3.0 [67] starting from ten random trees and taking the best tree obtained by Subtree-Pruning-Regrafting (SPR) search. The approximate likelihood ratio test (aLRT) was computed to perform a Shimodaira Hasegawa-like statistic to support every bifurcation. Based on the best-supported clades of these phylogenies, CNLs and TNLs were further divided into 20 and 46 homologous sequence groups (Additional file 1: Data 1), respectively. In the individual alignments for each of these sequence groups, the following aspects were checked: a) pairwise p-distances <0.5; b) third codon positions were not saturated and c) sequences that introduced gaps spanning over 25% of the length of the alignment were eliminated, as previously described [2]. Third codon position saturation was tested on fully resolved sites using DAMBE 5 [68]. These assessments yielded 9 CNL and 15 TNL groups with more than 5 sequences each, which were further used for analysis of molecular evolution, gene conversion and recombination (Table 1).

### DEFL sequences grouping and alignment

Our analyses of *Arabidopsis* DEFL sequences were based on those identified in the TAIR10 protein database by [29] and grouped in cysteine-rich peptide (CRP) groups [12]. In brief, CRPs were defined as proteins where the immature propeptide contains an N-terminal signal peptide for secretion (predicted by SignalP4.0 software), generally less than 170 amino acid residues in length and no less than 4 cysteine residues in its predicted mature sequence [69, 70]. From a previous CRP listing [17] we employed the complete coding sequences of DEFLs of *A. thaliana* assigned to 46 CRP groups CRP0000 to CRP1520 [29]. Sequences representing each of these DEFL groups and the criteria of length and number of cysteine residues previously mentioned were employed to identify further members from *A. thaliana* and those corresponding to *A. lyrata* and *A. halleri* using BLAST searches in Phytozome versions 10 and 11, between September 2015 and October 2016. Multiple sequence alignments with data from all three species were based on amino acid sequences with default settings in MUSCLE [71] implementation in SeaView 4.5.3 [72] and then manually corrected. Because alignments based on the CRP classification and including sequences from all three *Arabidopsis* species often reported pairwise *p*-distances >0.5 and third codon positions were saturated, they were further divided so the resulting alignments would be consistent with the previously described specifications employed for NLRs. Sequence group assessment generated a total of 156 alignment groups. From them, 47 with at least six sequences (Additional file 1: Data 1) were further employed for analysis of molecular evolution and gene recombination (Tables 1 and 3).

### Identifying genetic recombination

Geneconv [44], was used to detect recombination events between genes from the same species in the nucleotide alignments of the previously described groups of DEFLs, TNLs and CNLs. Global inner fragments were detected using a mismatch penalty (gscale = 2). The locations of significant global internal fragments with a Bonferroni-corrected Karlin-Altschul *p*-value ≤0.05 were parsed out, scaled to amino-acid residues and mapped on the protein domain structure of the longest or best characterized member from each sequence group using coordinates obtained from Uniprot (uniprot.org) or InterPro (ebi.ac.uk/interpro/protein).

GARD implemented in the Adaptive Evolution Server (datamonkey.org) was run as an alternative approach to detect recombination breakpoints in the alignment groups investigated with Geneconv. Program GARD searched each multiple sequence alignment for segment-specific phylogenies using an appropriate nucleotide substitution model identified with the Model Selection tool and default settings (no site-to-site rate variation and two rate classes) [35]. For alignment groups with evidences of recombination, GARD generates a list of significant breakpoints supported with a *p*-value ≤0.1.

Graphic representation of the results of Geneconv and GARD in Fig. 4 were drawn with the program IBS [73]. A scale based in codon/amino acid positions was employed to represent protein domains (amino acids), recombination events (nucleotides) and sites under selection (codons) in Fig. 4b and d.

### Analysis of molecular evolution

The ratio (ω) of the rate of nonsynonymous substitutions at nonsynonymous sites (dN) to synonymous (dS) substitutions at synonymous sites was estimated to figure out whether the coding region of a gene is under negative (purifying) selection (ω < 1), positive selection (ω > 1) or evolves neutrally (ω = 1). We analyzed the heterogeneity of selective pressures per codon sites in alignment groups of DEFLs, CNL and TNL genes with the program codeml from the PAML 4.8 package [48], running in the Athene1 computer cluster of the University of Regensburg. Based on the codon alignment and unrooted gene tree for each group, the models employed estimate ω and other parameters describing the pattern of codon substitution along sites. We investigated the occurrence of positive selection along codon sites by comparing nested model M2 with M1 and M8 with model M7. By comparing the likelihood of the model estimates with a Likelihood Ratio Test (LRT), codeml determines whether a model that considers positive selection fits the data better than one assuming neutral selection. The LRT statistics are assumed to be χ^2^ distributed with degrees of freedom equal to the difference in the number of parameters between models. The *p*-values of the likelihood ration test are corrected for multiple comparisons with the Benjamini-Hochberg procedure employing a False Discovery Rate of 0.10. M2 and M8 include a Bayes Empirical Bayes (BEB) analysis that detects codon sites under positive selection. We considered those with a posterior probability ≥0.9.

The second method to investigate the patterns of natural selection was FUBAR (Fast Unconstrained Bayesian AppRoximation) [49] implemented in the Adaptive Evolution Server (datamonkey.org). FUBAR takes the Neighbor-Joining tree or the trees inferred by GARD in the analysis of recombination (see previous section) and the alignment of a sequence group and determines the means of posterior distribution of synonymous (α) and non-synonymous (β) substitution rates over sites, as well as the mean posterior probability for ω > 1 or ω < 1 at a site. We reported the sites with evidence of pervasive positive (diversifying) or negative (purifying) at a posterior probability ≥0.9.

### Plant material and growth conditions

Seeds of *Arabidopsis lyrata* MN47 were surface sterilized, kept in sterile H_2_O at 4 °C for about 3 weeks in a horizontally placed Falcon vial. Seedlings were transferred to soil and grown under long-day conditions for two months. Plants at rosette stage were vernalized for 10 weeks at 4 °C. Subsequently flowering was induced by long-day conditions. Growth conditions of *A. thaliana* and *A. halleri* were described in [20].

### Infection with Fusarium graminearum

*F. graminearum* strain SG005/Fg005, an isolate from spring barley grain [74], was propagated and employed to infect *Arabidopsis* pistils as previously reported [20]. Inoculation medium for infection contains 1% Tween and conidia resuspended in sterile distilled water (final concentration of 8–9 × 10^5^ spores/ml). As described for *A. thaliana* and *A. halleri* [20], flowers of *Arabidopsis lyrata* were emasculated and allowed to recover for 24 h. Flowers and cauline leaves were inoculated by dipping them into conidial suspension for 20 min and then spraying with conidial solution. In order to favor development of *Fusarium* infection, inoculated plants were covered with a plastic bag sprayed with water and kept for 72 h under long-day conditions. After the period of infection, pistils and cauline leaves were collected in liquid nitrogen and stored at −80 °C. *Fusarium* infection was detected by staining pistils and leaves samples with wheat germ agglutinine-tetramethylrhodamine (WGA-TMR) following the protocol in [75]. Samples were analyzed using a confocal laser scanning microscope (LSM 510), excited by a 561 nm laser line, emission detected at 571 to 610 nm, respectively. Non-infected leaves and pistils collected 24 h after flower emasculation were employed in as control.

### RNA extraction, preparation of cDNA libraries and sequencing

As previously described for the analysis of *A. thaliana* and *A. halleri* samples [20], total RNA from infected and control *A. lyrata* pistil and leaf samples was extracted with the RNeasy Mini Plant Kit (Qiagen). After removal of residual genomic DNA, total RNA integrity and concentration were measured with a Bioanalyzer 2100 using the RNA 6000 Nano assay chip (Agilent Technologies). Library preparation and sequencing were carried out by the Center for Fluorescent Bioanalytics (KFB) at the University of Regensburg. Specifically, for cDNA library preparation the TruSeq RNA sample preparation kit (Illumina) was employed, starting from 500 ng of total RNA. Quantification of libraries was performed with the KAPA SYBR FAST ABI Prism Library Quantification Kit (Kapa Biosystems). Cluster generation with cBot (TruSeq PE Cluster Kit v3) was based on pooled equimolar amounts of each cDNA library. Sequencing was performed in a HiSeq 1000 instrument, using TruSeq SBS v3 reagents and the indexed paired-end (PE) protocol with 2 × 100 cycles. Image analyses and base calling, were converted into .fastq files with CASAVA 1.8.2. Library multiplexing was employed to obtain between 50 to 60 million reads per biological replicate, with a mean quality score of at least 37.

### RNA-seq analysis

Based on quality assessment with FastQC [76], reads were trimmed in the first and last 15 residues and subsequently mapped with CLC Genomics Workbench 7 (Qiagen) to the re-annotated *A. lyrata* reference genome v. 1 [77]. The following parameters were employed: mapping to genic and inter-genic regions, 10 maximum number of hits for a read, both strands, count paired reads as two, expression value as total counts, no global alignment, similarity fraction = 0.8, length fraction = 0.8, mismatch cost = 2, insertion cost = 3, deletion cost = 3. Identical settings were employed in the previously reported analysis of RNA-seq data from *A. thaliana* and *A. halleri* pistils [20].

Box-plots and Principal Component Analysis (PCA) were employed to assess variations in levels of expression between three biological replicates of each infected and non-infected pistil and leaf samples (data not shown). Results from the two most similar biological replicates were employed for further analyses of gene expression. The analysis was based on comparing read counts from infected tissue treatments with those of untreated samples, using the exact test for two-group comparisons from EdgeR [78] in the CLC Workbench. We classified as differentially expressed genes (DEGs) those with a false discovery rate-corrected *p*-value <0.0005, an expression fold change ≥2 for upregulation or ≤ −2 for downregulation. Only genes expressed with a RPKM ≥1 were considered.

## Additional files

Additional file 1: Data 1. Sheet 1. Overview of NLRs annotated in *A. thaliana*, *A. lyrata* and *A. halleri* and those aligned and included in gene trees to distinguish clades of CNLs and TNLs for further analyses of molecular evolution. Sheet 2. Lists of proteins annotated as NLRs in *A. thaliana*, *A. lyrata* and *A. halleri.* Note that in *A. lyrata* protein names and locus/gene names sometimes involve different IDs. Sheet 3. Groups of CNLs and TNLs of *A. thaliana*, *A. lyrata* and *A. halleri* identified in family gene trees. Sheet 4. Groups of *Arabidopsis* DEFLs. Those of *A. lyrata* and *A. halleri* were identified based on their similarity with those of *A. thaliana* previously annotated and assigned to specific CRP groups [12, 29] (XLSX 67 kb)

Additional file 2: Figure S1. Maximum likelihood gene tree of re-annotated complete and partial TNL genes from three *Arabidopsis* species. This gene tree was inferred from the alignment of coding sequences obtained with MAFFT, where only those reliably aligned columns with a GUIDANCE2 confidence score > 0.93 were employed. The most appropriate nucleotide substitution model was selected with program SMS and the maximum likelihood phylogenies was inferred with PhyML 3.0. The numbers on every node indicate posterior probabilities >0.70 supporting the phylogenetic relationships inferred. The clades employed for defining alignment groups (GT1 to GT46) are outlined with the first column of bars on the right of the gene tree. After evaluation of similarity levels and third codon position saturation, only sequences from groups indicated with orange bars where further investigated. Among them, clades indicated with ω + reported significant evidences of positive selection. This gene tree recovered the clades TNL-A to TNL-H identified in the first published phylogeny of *A. thaliana* TNLs [25], here they are indicated with a second column of bars on the right of the gene tree. Color-coding of sequence IDs indicates *Arabidopsis halleri* in green, *Arabidopsis lyrata* in black and *Arabidopsis thaliana* in red. The names of defense genes reported in the literature are indicated in parenthesis. (PDF 399 kb)

Additional file 3: **Data 2a.** Complete MAFFT alignment of CNLs. **Data 2b.** MAFFT alignment of CNLs containing only columns with a GUIDANCE2 confidence score > 0.93. (ZIP 103 kb)

Additional file 4: **Data 3a.** Complete MAFFT Alignment of TNLs. **Data 3b.** Complete MAFFT Alignment of TNLs containing only columns with a GUIDANCE2 confidence score > 0.93. (ZIP 393 kb)

Additional file 5: Data 4. Sequence statistics of groups analyzed for recombination and selection. (XLSX 52 kb)

Additional file 6: Figure S2. Gene trees of DEFLs with known species-specific or functional divergence. A Maximum Likelihood gene trees of LUREs in groups S1, S26 and S57. B Maximum Likelihood gene trees of PDF1s genes in groups S119 and S120. (DOCX 416 kb)

Additional file 7: Data 5. Results for all LRTs of NLR and DEFL families. (XLSX 52 kb)

Additional file 8: Figure S3. Study design and morphology of *Arabidopsis* pistils during infection with *Fusarium graminearum*. A Diagram describing the timeline for treatment and collection of *Arabidopsis* pistils and leaves employed for transcriptome profiling *Fusarium graminearum* infection. B Wheat germ agglutinine-tetramethylrhodamine (WGA-TMR) staining of *A. lyrata* mock-treated pistil showing that fungal hyphae are lacking inside the pistil. C WGA-TMR staining 3 days after infection (3DAI) of *A. lyrata* infected pistil showing *F. graminearum* hyphae. D WGA-TMR staining 3DAI of *A. lyrata* infected leaf showing *F. graminearum* hyphae growing on the epidermis of the leaf. Scale bars: 50 μm. (PPTX 888 kb)

Additional file 9: Data 6. Characteristics of the *Arabidopsis* transcriptomes compared*. (XLSX 54 kb)*

Additional file 10: Data 7. Differentially expressed genes in the transcriptome of *A. lyrata* pistils and leaves infected with *F. graminearum*. Expression data in RPKM units was employed to draw heat maps. (XLSX 761 kb)

Additional file 11: Figure S4. Heat map of TNL gene expression in untreated and *Fusarium* infected pistils and leaves in *Arabidopsis* species. All genes with average expression signals >1 RPKM in at least one condition are shown. Gene expression was scaled based on the distribution of all expression values for each gene family. White color denotes no expression, yellow means expression values in the 50th percentile (intermediate) and magenta denotes expression values in the 90th percentile (high). (PDF 238 kb)

Additional file 12: Figure S5. Heat map of DEFL gene expression in untreated and *Fusarium* infected pistils and leaves in *Arabidopsis* species. All genes with average expression signals >1 RPKM in at least one condition are shown. Gene expression was scaled based on the distribution of all expression values for each gene family. White color denotes no expression, yellow means expression values in the 50th percentile (intermediate) and magenta denotes expression values in the 90th percentile (high). (PDF 417 kb)

Additional file 13: Data 8. Sites under positive selection detected by M8 from codeml. Statistically significant are only those with a probabilitw ω>1= *: P>95%; **: P>99% Numbering is given according to a reference sequence and does not consider positions with indels. (XLS 166 kb)

## Abbreviations

AIC: Akaike information criterion
aLRT: Approximate likelihood ratio test
BEB: Bayes empirical Bayes analysis
CC: Coil-coil domain
CNL: Coil-coil-type NLR
CRP: Cysteine-rich peptide
DEFL: Defensin-like
DEGs: Differentially expressed genes
FUBAR: Fast unconstrained Bayesian approximation
GARD: Genetic algorithm for recombination detection
LCRs: Low-molecular weight, cysteine-rich
LRR: Leucine-rich repeat
LRT: Likelihood ratio test
ML: Maximum likelihood
NB-ARC: Nucleotide-binding adaptor shared by Apaf1, R genes and CED4
NLR: Nucleotide-binding domain and leucine-rich repeat containing protein also named as nucleotide-binding and oligomerization domain (NOD)-like receptor
PGIP: Polygalacturonase inhibitor proteins
PR: Pathogenesis-related gene or protein
RPKM: Reads per kilobase of transcript per million mapped reads
SCRLs: *S*-locus cysteine-rich-like
SPR: Subtree-prunning-regrafting
TIR: TOLL/interleukin1-receptor
TNL: TIR-type NLR
