# Supplementary figures and images for: Diversification of defensins and NLRs in Arabidopsis species by different evolutionary mechanisms
Source: BMC Evol Biol. 2017 Dec 15;17:255. doi: 10.1186/s12862-017-1099-4 (PMC5731061; doi:10.1186/s12862-017-1099-4)

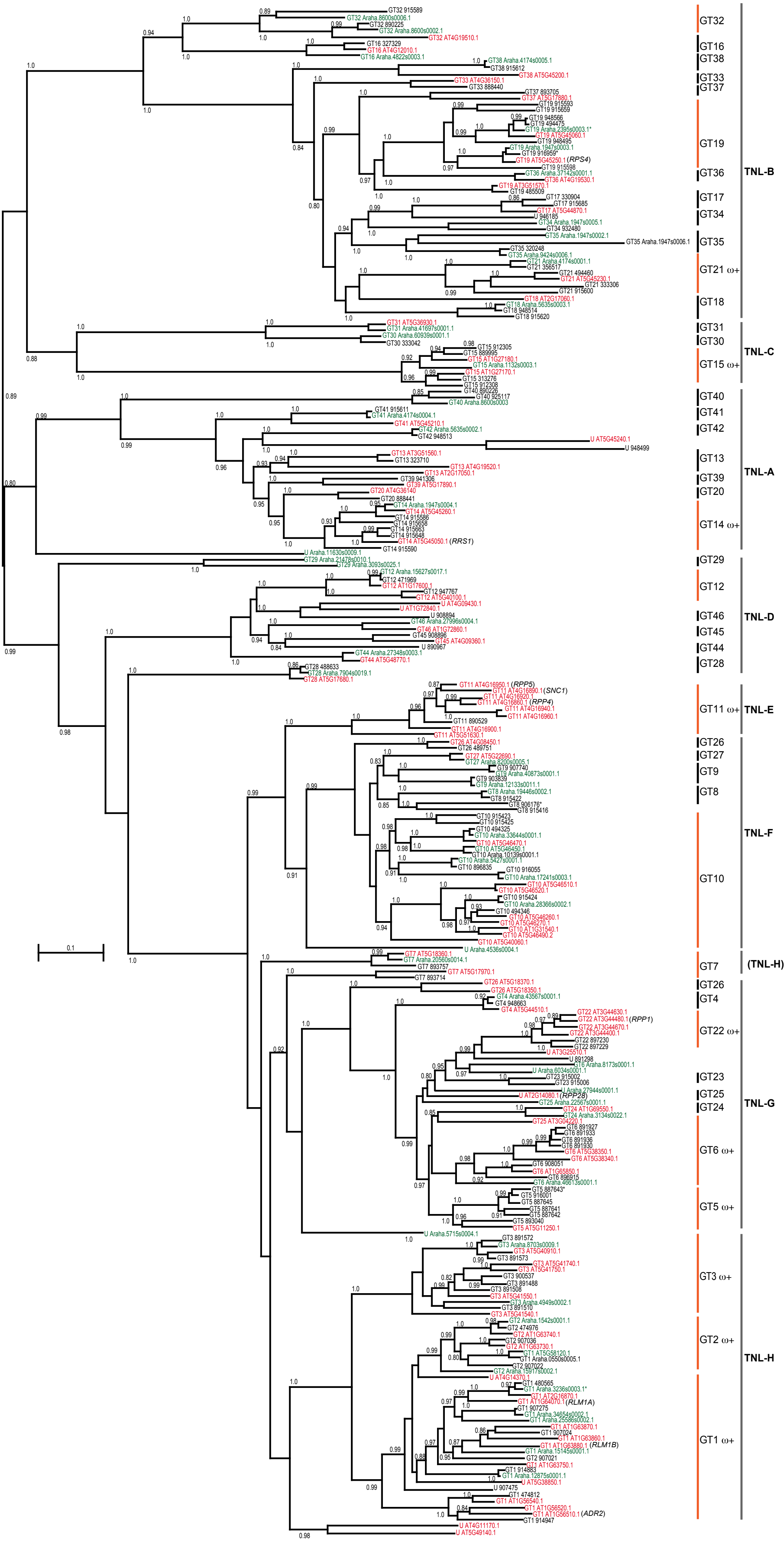

Supplement: Supplementary file 2 — Maximum likelihood gene tree of re-annotated complete and partial TNL genes from three Arabidopsis species. This gene tree was inferred from the alignment of coding sequences obtained with MAFFT, where only those reliably aligned columns with a GUIDANCE2 confidence score > 0.93 were employed. The most appropriate nucleotide substitution model was selected with program SMS and the maximum likelihood phylogenies was inferred with PhyML 3.0. The numbers on every node indicate posterior probabilities >0.70 supporting the phylogenetic relationships inferred. The clades employed for defining alignment groups (GT1 to GT46) are outlined with the first column of bars on the right of the gene tree. After evaluation of similarity levels and third codon position saturation, only sequences from groups indicated with orange bars where further investigated. Among them, clades indicated with ω + reported significant evidences of positive selection. This gene tree recovered the clades TNL-A to TNL-H identified in the first published phylogeny of A. thaliana TNLs [25], here they are indicated with a second column of bars on the right of the gene tree. Color-coding of sequence IDs indicates Arabidopsis halleri in green, Arabidopsis lyrata in black and Arabidopsis thaliana in red. The names of defense genes reported in the literature are indicated in parenthesis. (PDF 399 kb) [file 12862_2017_1099_MOESM2_ESM.pdf]
