# Supplementary material for: Diversification of defensins and NLRs in Arabidopsis species by different evolutionary mechanisms
Source: BMC Evol Biol. 2017 Dec 15;17:255. doi: 10.1186/s12862-017-1099-4 (PMC5731061; doi:10.1186/s12862-017-1099-4)
Supplement: Supplementary file 6 — Gene trees of DEFLs with known species-specific or functional divergence. A Maximum Likelihood gene trees of LUREs in groups S1, S26 and S57. B Maximum Likelihood gene trees of PDF1s genes in groups S119 and S120. (DOCX 416 kb) [file 12862_2017_1099_MOESM6_ESM.docx]

S26

**B**

S119

S57

S1

**A**

S120

Supplementary Figure 2
