# Supplementary material for: Diversification of defensins and NLRs in Arabidopsis species by different evolutionary mechanisms
Source: BMC Evol Biol. 2017 Dec 15;17:255. doi: 10.1186/s12862-017-1099-4 (PMC5731061; doi:10.1186/s12862-017-1099-4)
Supplement: Supplementary file 8 — Study design and morphology of Arabidopsis pistils during infection with Fusarium graminearum. A Diagram describing the timeline for treatment and collection of Arabidopsis pistils and leaves employed for transcriptome profiling Fusarium graminearum infection. B Wheat germ agglutinine-tetramethylrhodamine (WGA-TMR) staining of A. lyrata mock-treated pistil showing that fungal hyphae are lacking inside the pistil. C WGA-TMR staining 3 days after infection (3DAI) of A. lyrata infected pistil showing F. graminearum hyphae. D WGA-TMR staining 3DAI of A. lyrata infected leaf showing F. graminearum hyphae growing on the epidermis of the leaf. Scale bars: 50 μm. (PPTX 888 kb) [file 12862_2017_1099_MOESM8_ESM.pptx]

## Slide 1
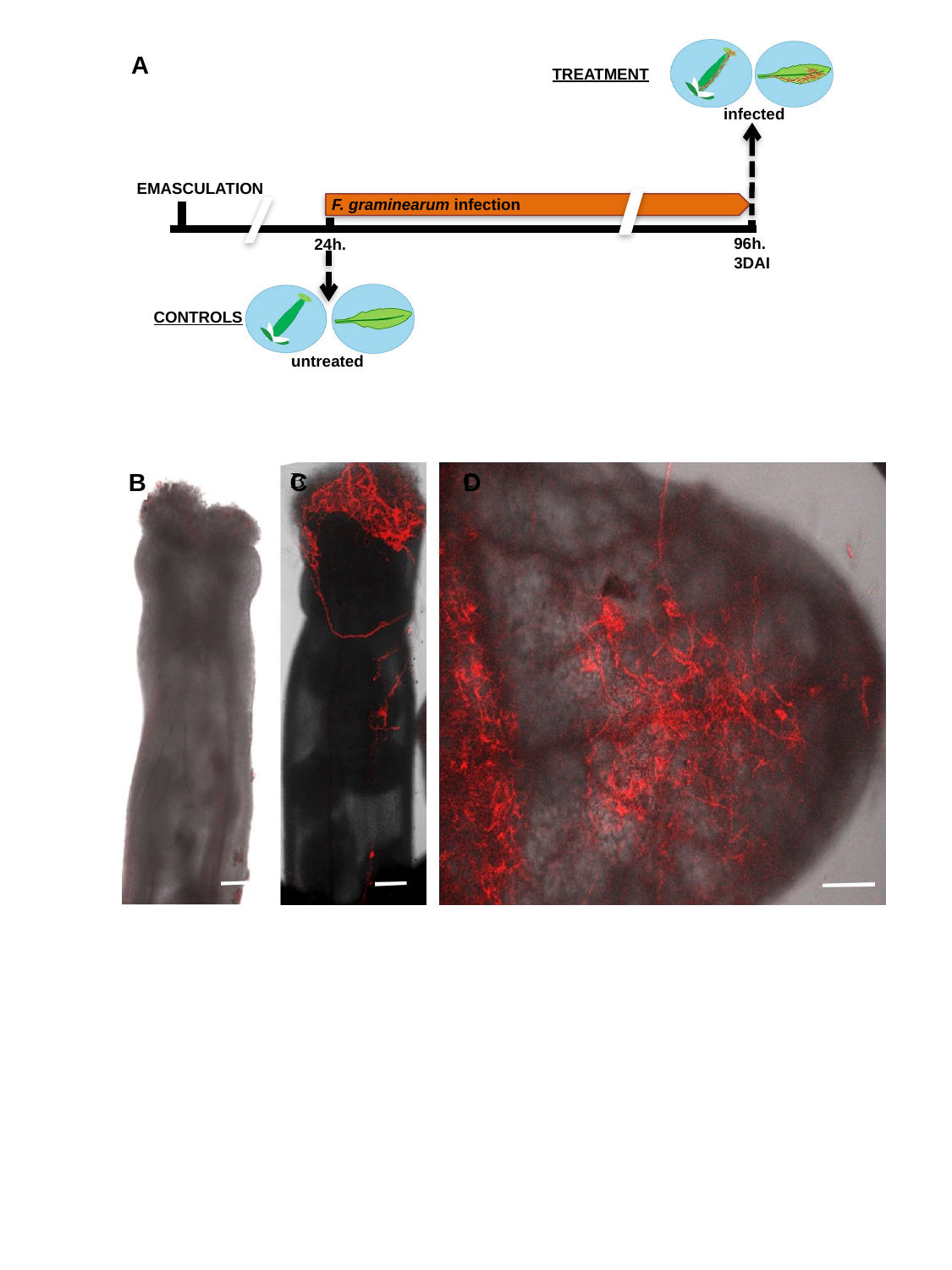

A
TREATMENT
infected
EMASCULATION
F. graminearum infection
96h.
3DAI
24h.
CONTROLS
untreated
B
C
D
