# Supplementary material for: Diversification of defensins and NLRs in Arabidopsis species by different evolutionary mechanisms
Source: BMC Evol Biol. 2017 Dec 15;17:255. doi: 10.1186/s12862-017-1099-4 (PMC5731061; doi:10.1186/s12862-017-1099-4)
Supplement: Supplementary file 11 — Heat map of TNL gene expression in untreated and Fusarium infected pistils and leaves in Arabidopsis species. All genes with average expression signals >1 RPKM in at least one condition are shown. Gene expression was scaled based on the distribution of all expression values for each gene family. White color denotes no expression, yellow means expression values in the 50th percentile (intermediate) and magenta denotes expression values in the 90th percentile (high). (PDF 238 kb) [file 12862_2017_1099_MOESM11_ESM.pdf]

Alignment group	Gene ID	Untreated pistils	Infected pistils	Untreated leaf	Infected leaf
GT1	907024				
	907275				
	Araha.0550s0005				
	Araha.12875s0001				
	Araha.15145s0001				
	Araha.25586s0002				
	Araha.3236s0003				
	Araha.34654s0002				
	AT1G56510				
	AT1G56520				
	AT1G56540				
	AT1G63750				
	AT1G63860				
	AT1G63880				
	AT2G16870				
	AT5G58120				
GT2	474976				
	907022				
	907036				
	Araha.1542s0001				
	Araha.15917s0002				
	AT1G63730				
	AT1G63740				
GT3	891488				
	891508				
	891510				
	891572				
	900537				
	Araha.4949s0002				
	Araha.8703s0009				
	AT5G40910				
	AT5G41550				
	AT5G41740				
	AT5G41750				
GT4	948663				
	Araha.43567s0001				
	AT5G44510				
GT5	887645				
	893040				
	AT5G11250				
GT6	896915				
	Araha.46613s0001				
	Araha.8173s0001				
	AT5G38350				
GT7	893757				
	Araha.20560s0014				
	AT5G18360				
GT8	915416				
	Araha.19446s0002				
GT9	Araha.12133s0011				
	Araha.40873s0001				
GT10	494325				
	494346				
	896835				
	915423				
	915424				
	Araha.10139s0001				
	Araha.17241s0003				
	Araha.28366s0002				
	Araha.33644s0001				
	Araha.5427s0001				
	AT1G31540				
	AT5G40060				
	AT5G46260				
	AT5G46270				
	AT5G46450				
	AT5G46470				
	AT5G46490				
	AT5G46510				
	AT5G46520				
GT11	890529				
	AT4G16860				
	AT4G16890				
	AT4G16900				
	AT4G16920				
	AT4G16940				
	AT4G16950				
	AT4G16960				
	AT5G51630				
GT12	471969				
	947767				
	Araha.15627s0017				
	AT1G17600				
	AT5G40100				
GT13	323710				
	AT4G19520				
GT14	915663				
	Araha.1947s0004				
	AT5G45050				
	AT5G45260				
GT15	313276				
	912308				
	Araha.1132s0003				
	AT1G27170				
	AT1G27180				
GT16	327329				
	Araha.4822s0003				
	AT4G12010				
GT17	915685				
	AT5G44870				
GT18	915620				
	Araha.5635s0003				
GT19	485509				
	915593				
	916959				
	Araha.1947s0003				
	Araha.2395s0003				
	AT5G45060				
	AT5G45250				
GT20	888441				
	AT4G36140				
GT21	356517				
	494460				
GT22	897230				
	AT3G44400				
	AT3G44480				
	AT3G44630				
	AT3G44670				
GT24	Araha.3134s0022				
	AT1G69550				
GT25	Araha.22567s0001				
	AT3G04220				
GT26	AT5G18350				
	AT5G18370				
GT27	Araha.8200s0005				
	AT5G22690				
GT28	488633				
	Araha.7904s0019				
GT29	Araha.21478s0010				
	Araha.3093s0025				
GT30	Araha.60939s0001				
GT31	Araha.41697s0001				
	AT5G36930				
GT32	915589				
	Araha.8600s0002				
	Araha.8600s0006				
	AT4G19510				
GT33	AT4G36150				
GT34	932480				
	Araha.1947s0005				
GT35	320248				
	Araha.1947s0002				
	Araha.1947s0006				
	Araha.9424s0006				
GT36	Araha.37142s0001				
	AT4G19530				
GT37	893705				
	AT5G17880				
GT39	941306				
	AT5G17890				
GT40	Araha.8600s0003				
GT42	948513				
GT44	AT5G48770				
GT45	908896				
GT46	AT1G72860				
Ungrouped	890967				
	891298				
	894148				
	908894				
	946185				
	948499				
	Araha.27944s0001				
	Araha.5715s0004				
	Araha.6034s0001				
	AT1G72840				
	AT2G14080				
	AT3G25510				
	AT4G09430				
	AT4G11170				
	AT4G14370				
	AT5G38850				
	AT5G45240				
